# Supplementary material for: Role of Klotho as a Modulator of Oxidative Stress Associated with Ovarian Tissue Cryopreservation
Source: Int J Mol Sci. 2021 Dec 17;22(24):13547. doi: 10.3390/ijms222413547 (PMC8707502; doi:10.3390/ijms222413547)
Supplement: Supplementary file 1 [file ijms-22-13547-s001.zip › ijms-1470364-supplementary.pdf]

Supplementary Figure S1.

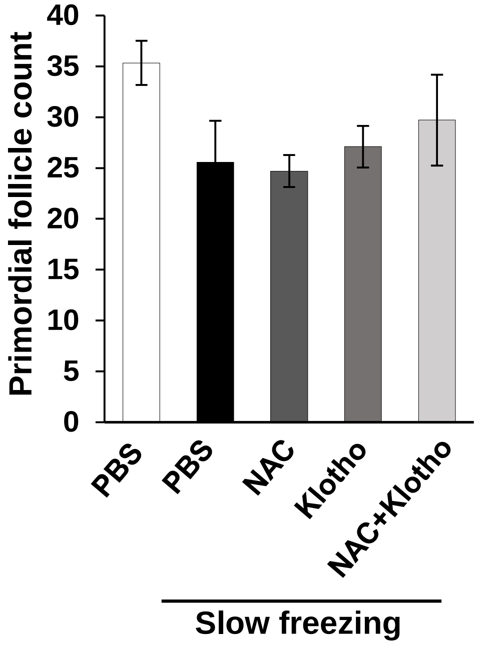

Supplementary Figure S1. Slow-freezing cryopreservation reveals changes in primordial follicle number. Primordial follicle density in frozen–thawed ovarian tissues. There was no significant differences in follicle density between each group.

Supplementary Figure S2.

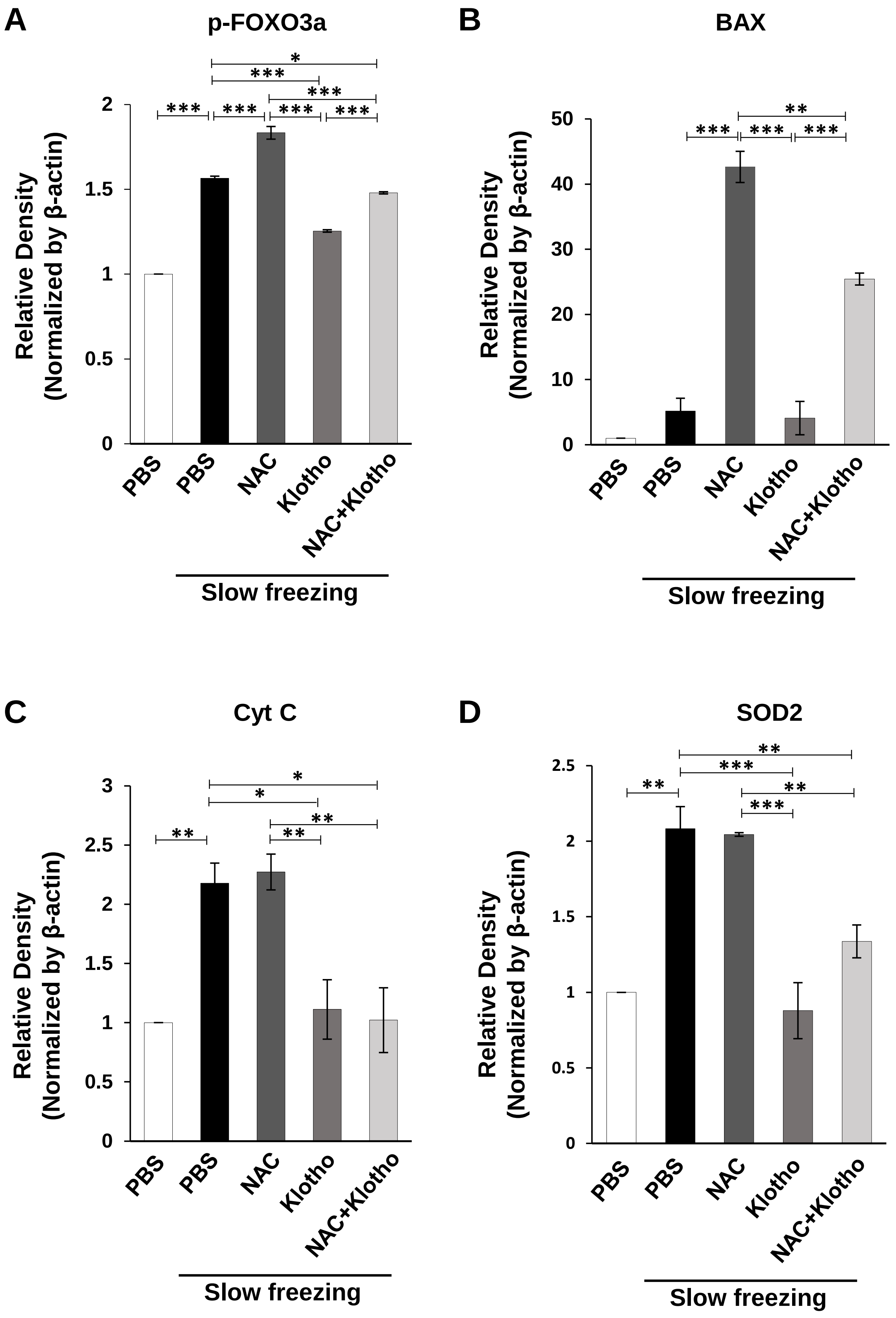

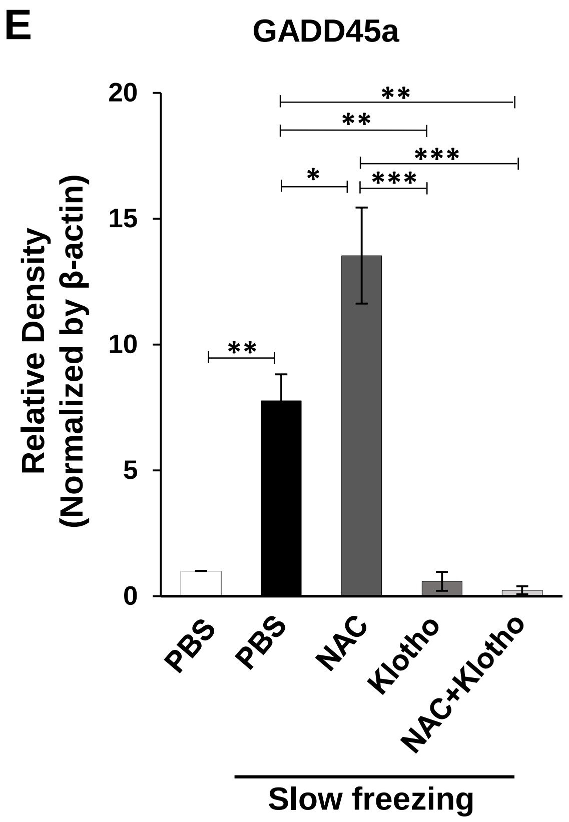

Supplementary Figure S2. Klotho reduced NAC-induced apoptosis. (A-E) Immunoblotting quantification by densitometry analyzed by ImageJ in Figure 4C. Each image was normalized by β-actin loading control. All data are shown as mean ± SD. The p values were determined by one-way ANOVA. *, $p < 0.05$; **, $p < 0.01$; ***, $p < 0.001$.
